# Physiological and Psychological Effects of Viewing a Kiwifruit (*Actinidia deliciosa* ‘Hayward’) Orchard Landscape in Summer in Japan

**DOI:** 10.3390/ijerph120606657

**Published:** 2015-06-11

**Authors:** Miho Igarashi, Masayuki Miwa, Harumi Ikei, Chorong Song, Michiko Takagaki, Yoshifumi Miyazaki

**Affiliations:** 1 Center for Environment, Health and Field Sciences, Chiba University, 6-2-1 Kashiwa-no-ha, Kashiwa, Chiba 277-0882, Japan; E-Mails: miho.murachi@gmail.com (M.I.); mmiwa@faculty.chiba-u.jp (M.M.); crsong1028@gmail.com (C.S.); mtgaki@faculty.chiba-u.jp (M.T.); 2 Forestry and Forest Products Research Institute, 1 Matsunosato, Tsukuba, Ibaraki 305-8687, Japan; E-Mail: ikei0224@ffpri.affrc.go.jp

**Keywords:** Japanese urban agriculture, kiwifruit orchard, summer, physiological relaxation, psychological relaxation

## Abstract

The physiological and psychological relaxation effects of viewing a kiwifruit (*Actinidia deliciosa* ‘Hayward’) orchard landscape were investigated. Seventeen Japanese adult females (46.1 ± 8.2 years) viewed a kiwifruit orchard landscape or a building site (control) for 10 min. The heart rate variability and heart rate were determined. The modified semantic differential method and the short-form Profile of Mood States were used to assess the psychological effects. Compared with viewing the building site, viewing the kiwifruit orchard landscape resulted in a significant increase in the parasympathetic activity, a marginally significant decrease in the heart rate, a significant increase in “comfortable”, “relaxed” and “natural” feelings and a significant improvement in mood states.

## 1. Introduction 

In modern society, people are exposed to various stressors [1,2], and many people empirically know that contact with nature decreases stress and leads to a more relaxed state. The relaxation effect of viewing nature has been the subject of psychophysiological studies since the 1990s [3,4,5]. From a preventive medical standpoint, forest therapy is believed to improve immunocompetence through plant-derived physiological relaxation [6]. In previous forest therapy studies, many physiological indices, such as the heart rate to assess autonomic nerve activity (assessed by heart rate variability (HRV) and blood pressure) [7,8,9,10,11,12], prefrontal cortex activity (assessed by near-infrared spectroscopy) [13] and endocrine activity (assessed by salivary cortisol) [7,8,9,11,12,13,14,9,11], were measured. The results of these previous studies have shown physiological relaxation effects associated with forest environments and that contact with nature was physiologically beneficial. However, it may be difficult for some urban inhabitants to go to a large forest because of the lack of opportunity and/or time. Nonetheless, many people wish to have intimate contact with the natural environment. 

Recently, among the inhabitants of developed countries, interest in food has increased, and there has been a tendency of enjoying contact with nature and increased attention regarding allotment gardens [15]. However, these findings were obtained using questionnaires, and few studies have investigated the physiological relaxation effects of interactions with gardening [16]. 

We developed an interest in evaluating the physiological and psychological effects of viewing kiwifruit (*Actinidia deliciosa* ‘Hayward’) orchards on humans at a university in Chiba Prefecture, Japan. Chiba Prefecture is a commuter town located near Tokyo that has become urbanized. However, agriculture is still actively practiced in the community. The aim of the present study was to investigate (1) the physiological relaxation effects of viewing a kiwifruit orchard landscape on autonomic nerve activity by measuring HRV and heart rate and (2) psychological relaxation effects as assessed by the modified semantic differential (SD) method and the short-form Profile of Mood States (POMS).

## 2. Experimental Section 

### 2.1. Experimental Areas

**Figure 1:**
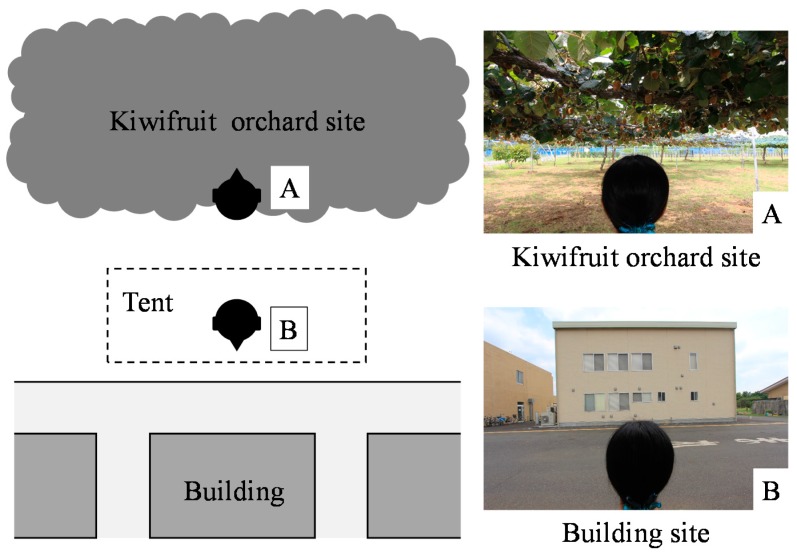
Schematic and photographs showing the two scenes that were viewed.

The field experiment was performed in the summer (August) of 2013 at the Center for Environment, Health and Field Sciences, Chiba University in Chiba, Japan. The center can be reached in approximately 30 min by train from Tokyo. The kiwifruit orchard was viewed from the edge of the orchard. The orchard site was located close to the building site (approximately 15 m); if subjects turned around, they could view the opposite area (Figure 1). At each experimental site, viewing spots were located outdoors. At the orchard site, there were 14 kiwifruit trees (*A. deliciosa* ‘Hayward’) bearing many fruits. Subjects avoided direct sunlight because of the kiwifruit leaves. At the building site, there was a two-story building and a well-paved road. In addition, subjects avoided direct sunlight by taking shelter in a tent. 

The experimental conditions were controlled so that they were similar at both the orchard site and the building site. The weather on the day of the experiment was sunny, and the average temperature, humidity and intensity of illumination in the orchard site were 32.9 °C, 59.8% and 2020 lx, respectively, and those of the building site were 33.1 °C, 58.8% and 9580 lx, respectively. 

### 2.2. Subjects

Seventeen Japanese adult females were recruited. Subjects lived in urban areas in the suburbs of Kashiwa in the Chiba Prefecture; none of them were farmers. None of the subjects reported having any physiological or psychiatric disorders in their personal histories. In addition, none of the subjects were menstruating on the day of the experiment. The adults who finally participated in the study had a mean age of 46.1 ± 8.2 years and a mean body mass index of 21.4 ± 2.0 kg/m^2^; those who had smoking or drinking habits were excluded. All subjects agreed to the study protocol and signed a written informed consent. This study was conducted under the regulations of the Ethics Committee of the Center for Environment, Health and Field Sciences, Chiba University, Japan.

### 2.3. Procedure

**Figure 2:**
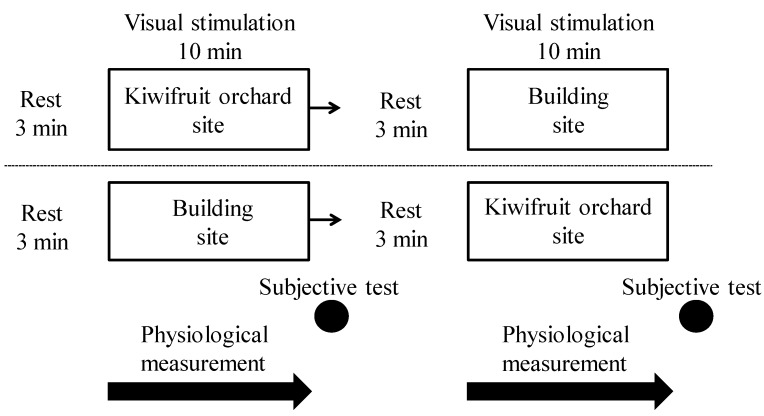
Study protocol.

Seventeen subjects were randomly divided into five groups of four or one, which eliminated the ordering effect. Each group’s experiment started at hourly intervals. Subjects assembled in the waiting room 30 min before their start time. After orientation regarding the experiment, subjects took approximately 1 min to move to their assigned spots for viewing either the landscape or the building site. Subjects then took approximately 3 min to rest while sitting in a camping chair and then viewed the landscape or building for 10 min quietly in the camping chair. The physiological index was measured continuously during the 10-min simulation (Figure 2). After the physiological and psychological measurements, subjects repeated the process to view the other scene. 

### 2.4. Physiological Measurement

As a physiological measurement, HRV was analyzed for periods between consecutive R waves in the electrocardiogram (RR intervals), as measured by a portable electrocardiograph (Activtracer, AC-301A; GMS) [17,18]. This device uses a 3-lead electrocardiogram (Lead II) to perform measurements. The power levels of the high frequency (HF) (0.15–0.40 Hz) and low frequency (LF) (0.04–0.15 Hz) components of HRV were calculated by the maximum entropy method (MemCalc/Win; GMS). The HF power was considered to reflect the parasympathetic nerve activity, and the LF/HF power ratio was determined to reflect the sympathetic nerve activity [19,20]. In this study, the natural logarithmic value of the HF (lnHF) power or the LF/HF power ratio (lnLF/HF) was used [21]. The heart rate and HRV data were collected for the entire 10 min. 

### 2.5. Psychological Measurement

Two different questionnaires were used to investigate psychological responses. The questionnaires were completed after viewing at each experimental site. The evaluation by the modified SD method [22] was performed using three pairs of adjectives on 13 scales, including “comfortable—uncomfortable,” “relaxed—awakening” and “natural—artificial.” In addition, each participant filled out the POMS [23,24,25] questionnaire, which assesses six mood states: tension–anxiety (T–A), depression (D), anger–hostility (A–H), fatigue (F), confusion (C) and vigor (V). In the POMS test, a short form with 30 questions was used to decrease the burden on subjects.

### 2.6. Statistical Analysis

All data are shown as the means ± standard errors. Physiological and psychological tests were used to compare the orchard and building areas. A paired *t*-test was used to compare differences in mean physiological data over a period of 10 min while viewing the orchard site or the building site. The Wilcoxon signed-rank test was used to analyze differences in psychological indices after viewing two environments. Statistical analysis was performed using SPSS 21.0 (IBM Corp., Armonk, NY, USA). A one-sided test was used in this study because of the hypothesis that humans would be relaxed by viewing a kiwifruit orchard landscape. In all cases, values of *p* < 0.05 were considered to indicate statistical significance.

## 3. Results

### 3.1. Physiological Effects

**Figure 3:**
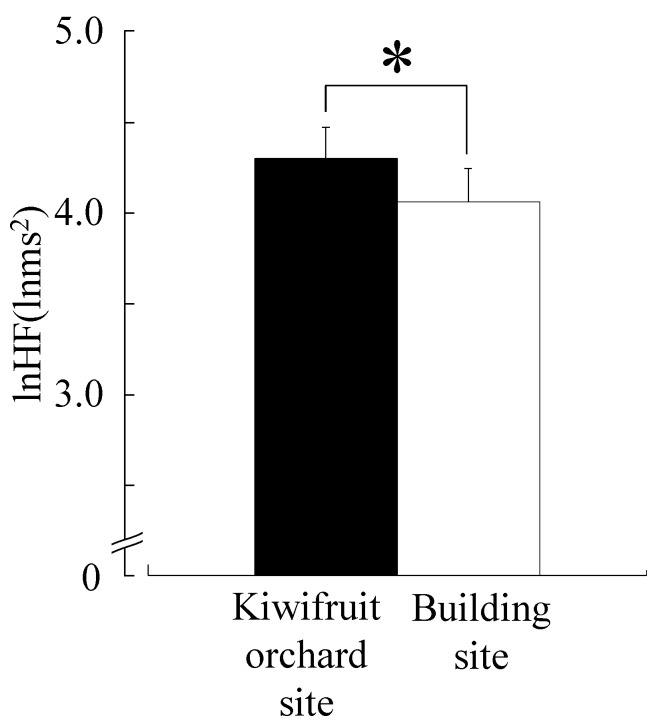
Comparison of the natural logarithmic value of high-frequency (HF) power levels of the heart rate variability during 10-min viewing of a kiwifruit orchard or a building site. Data are expressed as the means ± standard error (SE); n = 17. * *p* < 0.05 by the paired *t*-test (one-sided).

**Figure 4:**
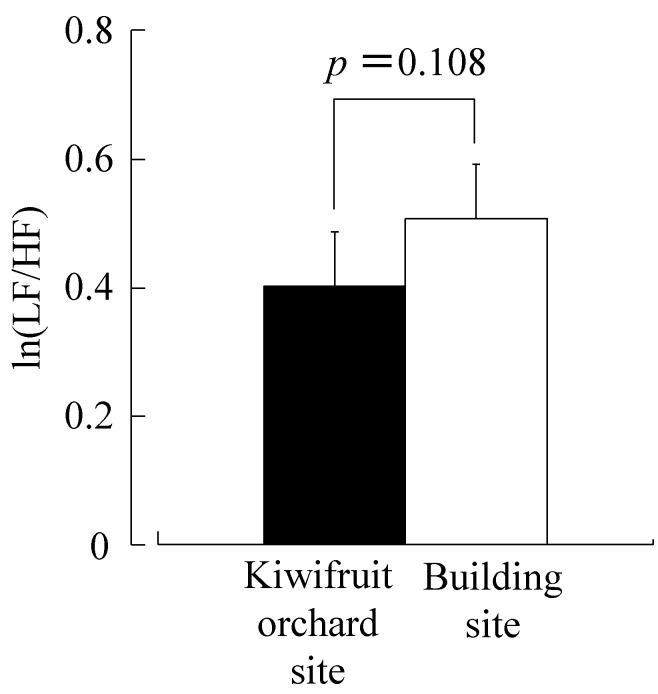
Comparison of the natural logarithmic value of the low frequency (LF)/high frequency power ratio of heart rate variability during 10-min viewing of kiwifruit orchard or a building site. Date are expressed as the means ± standard error (SE); n = 17. *p*-value by the paired *t*-test (one-sided).

**Figure 5:**
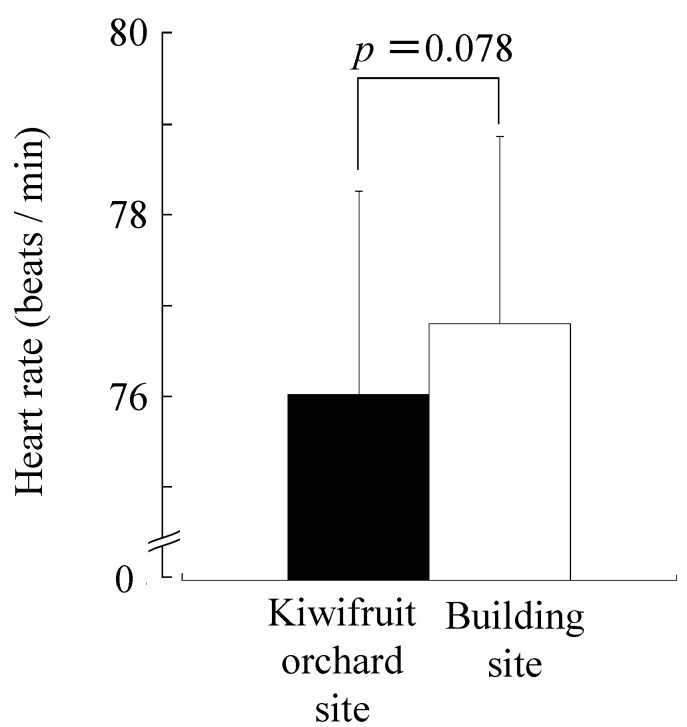
Comparison of the heart rate during 10-min viewing of a kiwifruit orchard or a building site. Data are expressed as the means ± SE; n = 17. *p*-value by the paired *t*-test (one-sided).

Figure 3 shows lnHF, which is considered to reflect the parasympathetic nerve activity for 10 min. The lnHF value of the kiwifruit site (4.30 ± 0.17 lnms^2^) was significantly higher than that of the building site (4.06 ± 0.18 lnms^2^, *p* < 0.05). With regard to the ln(LF/HF) ratio that is considered to reflect the sympathetic nerve activity for 10 min, the kiwifruit site (0.40 ± 0.08) was lower than the building site (0.51 ± 0.08), but the difference was not statistically significant (Figure 4). Figure 5 shows the heart rate for 10 min. The heart rate of the kiwifruit site (76.0 ± 2.2 bpm) was marginally significantly lower than that of the building site (76.8 ± 2.1 bpm, *p* < 0.10).

### 3.2. Psychological Effects

**Figure 6:**
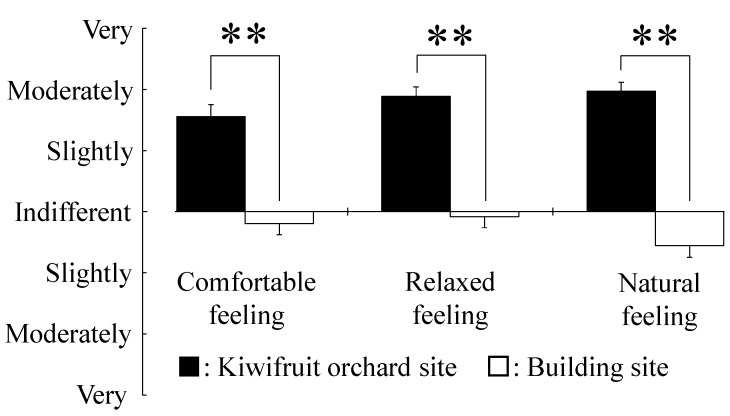
Comparison of the semantic differential questionnaire after 10-min viewing of a kiwifruit orchard or a building site. Data are expressed as the means ± SE; n = 17. ** *p* < 0.01 by the Wilcoxon signed-rank test (one-sided).

Figure 6 shows the results of the modified SD method for “comfortable,” “relaxed” and “natural” feelings by viewing the kiwifruit orchard or the building site. The kiwifruit site was rated from slightly “comfortable,” “relaxed” and “natural” to moderately “comfortable,” “relaxed” and “natural,” and the building site was rated from indifferent to slightly “uncomfortable,” “awakening” and “artificial” (*p* < 0.01).

**Figure 7:**
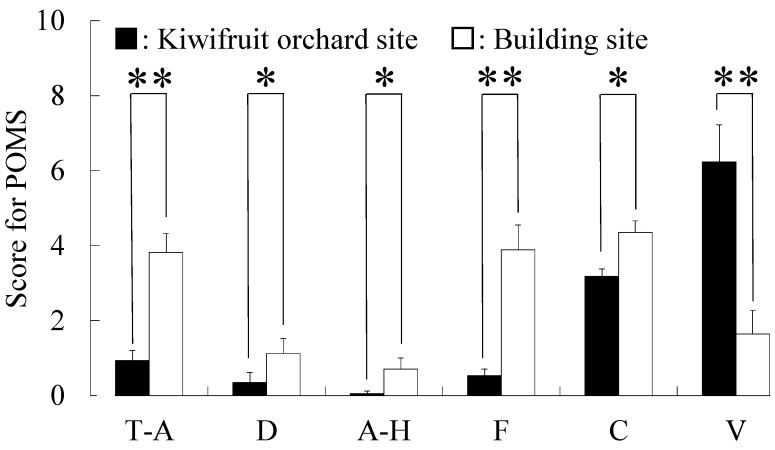
Comparison of Profile of Mood States (POMS) questionnaire results after 10-min viewing of a kiwifruit orchard site or a building site. T–A, tension–anxiety; D, depression; A–H, anger–hostility; F, fatigue; C, confusion; V, vigor. Data are expressed as the means ± SE; n = 17. * *p* < 0.05, ** *p* < 0.01 by the Wilcoxon signed-rank test (one-sided).

In addition, differences were detected in the POMS short test (Figure 7), with scores for the negative subscales of T–A (*p* < 0.01), D (*p* < 0.05), A–H (*p* < 0.05), F (*p* < 0.01) and C (*p* < 0.05) being significantly lower after viewing the kiwifruit site than corresponding scores after viewing the building site. Conversely, the positive mood state V was significantly higher after viewing the kiwifruit site than after viewing the building site (*p* < 0.01).

## 4. Discussion

Viewing a kiwifruit orchard landscape resulted in a significant increase in the parasympathetic nerve activity and a marginally significant decrease in the heart rate relative to those after viewing the building site. The findings indicated that 10-min viewing of the kiwifruit orchard landscape induced physiological relaxation. The duration of the viewing period was limited to 10 min because some subjects felt sleepy after longer sessions. In previous studies, the relaxation effects of viewing a forest while being in a sitting position relative to those viewing an urban scene were shown. These studies reported a decreased pulse rate or heart rate in the autonomic nerve activity [7,9,10,11,14], increased parasympathetic nerve activity [7,9,10,11,12,26] and decreased sympathetic nerve activity [7,9,10,11,12,26], blood pressure [9,10,12,14] and salivary cortisol levels [7,9,11,12,13,14]. Furthermore, a study that compared walking in a forest with walking in an urban setting reported decreased pulse rate or heart rate in the autonomic nerve activity [9,10,11,27], increased parasympathetic nerve activity [7,9,10,11,12] and decreased sympathetic nerve activity [7,9,10,11], blood pressure [9,10] and salivary cortisol levels [9,11,12]. The present study results showing increased parasympathetic nerve activity and decreased heart rate are in accordance with these previous results for forest *vs.* urban viewing. Furthermore, two studies reported that walking in a park induced increased parasympathetic nerve activity and decreased heart rate, which is also in accordance with the results of the present study [28,29].

In addition, some studies clarified beneficial effects of forest therapy in immune function in subjects who had physiological stress [30,31,32]. Subjects had low natural-killer (NK) cell activity before the experiment, but NK cell activity increased after forest therapy for three days and two nights. It was reported that immune function improved, and improvement effects continued for one month. 

From the results of psychological effects, the response to the kiwifruit orchard site was perceived as being more “comfortable,” “relaxed” and “natural.” The present study results are in accordance with those of previous studies conducted in forests [10,12,14,33] and urban parks [27,28]. The main previous relaxation studies of horticultural products used the questionnaire evaluation. There have been a few reports regarding physiological relaxation effects [16,34]. However, van den Berg and Custers [16] used indoor reading as a control, which we considered inappropriate. In the study by Lee *et al.* [34], there were no significant differences among subjects, and the authors indicated significant differences in cases of classification by personality, which we think was a limitation in that study. In evaluations of various horticultural therapies, physiological evaluations, such as HRV and heart rate, will be primarily required in the future from the perspective of evidence-based medicine; however, psychological evaluations should also be performed.

In the short-form POMS test, scores for the negative subscales of T–A, D, A–H, F and C were significantly lower after viewing the kiwifruit orchard than those after viewing the building site, and the positive mood state V was significantly higher after viewing the kiwifruit orchard than after viewing the building site. Wichrowski *et al.* [35] reported on horticultural therapy for rehabilitation of patients with heart diseases, and the results of this study were also in accordance with those of the present study. A comparison of the effects of viewing a forest or an urban area have also been reported [7,32], and the results were also in accordance with those of the present study. 

The physiological and psychological relaxation effects associated with viewing a kiwifruit orchard were clearly shown in this study, and potential applications in stress management are apparent. These findings, combined with the known nutritional effects of kiwifruit, could be important horticultural considerations for future orchard usage in modern stressful societies. Urban landscape planning involving fruit trees is believed to bring a sense of relaxation to inhabitants. If the effects of introducing fruit trees to the urban landscape on physiological relaxation become clear, these may contribute to improve the quality of life for urban inhabitants.

In this study, the effects of viewing a kiwifruit orchard in summer on 30–50-year-old female subjects were investigated. This study has some limitations. First, this study compared the impact of viewing kiwifruit orchards and buildings. Therefore, future studies should compare the physiological parameters of each subject measured before and after the viewing. Second, the subject group should cover both genders and a wide range of ages to determine whether the relaxation effect of viewing a kiwifruit orchard landscape on the nervous system applies to the general population. Third, other studies should compare the relaxation potency of different natural environments.

## 5. Conclusions

Compared with viewing a building site, viewing a kiwifruit orchard landscape resulted in a significant increase in the parasympathetic nerve activity assessed by lnHF, a marginally significant decrease in heart rate, a significant increase in “comfortable,” “relaxed” and “natural” feelings assessed by the modified SD method and significant improvements in mood states assessed by POMS. The findings indicate that viewing a kiwifruit orchard landscape for 10 min while sitting in a chair induced physiological and psychological relaxation.
